# Supplementary material for: SARS-CoV-2 antibody dynamics and transmission from community-wide serological testing in the Italian municipality of Vo’
Source: Nat Commun. 2021 Jul 19;12:4383. doi: 10.1038/s41467-021-24622-7 (PMC8289856; doi:10.1038/s41467-021-24622-7)
Supplement: Supplementary file 1 — Supplementary Information [file 41467_2021_24622_MOESM1_ESM.pdf]

Supplementary Information

SARS-CoV-2 antibody dynamics and transmission from community-wide serological testing in the Italian municipality of Vo'

Ilaria Dorigatti[1^*], Enrico Lavezzo[2^*], Laura Manuto[2], Constanze Ciavarella[1], Monia Pacenti[3], Caterina Boldrin[3], Margherita Cattai[3], Francesca Saluzzo[2], Elisa Franchin[2,], Claudia Del Vecchio[2,], Federico Caldart[2], Gioele Castelli[2], Michele Nicoletti[2], Eleonora Nieddu[2], Elisa Salvadoretti[2], Beatrice Labella[2], Ludovico Fava[2], Simone Guglielmo[2], Mariateresa Fascina[2], Marco Grazioli[2], Gualtiero Alvisi[2], Maria Cristina Vanuzzo[3], Tiziano Zupo[3], Reginetta Calandrin[3], Vittoria Lisi[3], Lucia Rossi[3], Ignazio Castagliuolo[2], Stefano Merigliano[4], H. Juliette T. Unwin[1], Mario Plebani[5], Andrea Padoan[5], Alessandra R. Brazzale[6], Stefano Toppo[2,7], Neil M. Ferguson[1], Christl A. Donnelly[1,8], Andrea Crisanti[2,3,9*]

[1]MRC Centre for Global Infectious Disease Analysis and the Abdul Latif Jameel Institute for Disease and Emergency Analytics (J-IDEA), School of Public Health, Imperial College London, London, UK
[2]Department of Molecular Medicine, University of Padova, Padova, Italy
[3]Azienda Ospedale Padova, Via Giustiniani 1, Padova, Italy
[4]Department of Surgery, Oncology and Gastroenterology, University of Padova, Padova, Italy
[5]Department of Medicine, University of Padova, Padova, Italy
[6]Department of Statistical Sciences, University of Padova, Padova, Italy
[7]CRIBI Biotech Centre, University of Padova, Padova, Italy
[8]Department of Statistics, University of Oxford, Oxford, UK
[9]Department of Life Science Imperial College London, Exhibition Road, London, UK

[^]Equal contributions

[*]Corresponding authors
Dr Ilaria Dorigatti (i.dorigatti@imperial.ac.uk)
Prof Enrico Lavezzo (enrico.lavezzo@unipd.it)
Prof Andrea Crisanti (andrea.crisanti@unipd.it; a.drcrisanti@imperial.ac.uk)

Contents

Supplementary Methods

Section 1	Serological test details	3
Section 2	Contact and ground truth definitions	4
Section 3	Seroprevalence estimates details	4
Section 4	Within-household transmission model	5
Section 5	SARS-CoV-2 transmission model with contact tracing	6

Supplementary Note 1

8

Supplementary Figures

Figure 1	Association among serological and micro-neutralisation assays	9
Figure 2	Household size distributions, number of infections and attack rates by household size	10
Figure 3	Within-household transmission models performance metric	11
Figure 4	Flow diagram of the transmission model used to estimate the impact of contact tracing	11

Supplementary Tables

Table 1	SARS-CoV-2 exposure authentication criteria	12
Table 2	Observed test results combinations in the May and November 2020 serosurveys	12
Table 3	Assay-specific performance against the different ground truth definitions	13
Table 4	Positive (+) Serological results on the subjects tested with all three assays in May and November 2020.	13
Table 5	Association analysis on antibody titres	14
Table 6	Association analysis on antibody decay rates	14
Table 7	Frequency of comorbidities in symptomatic and asymptomatic SARS-CoV-2 infected individuals	14
Table 8	Frequency of medication type in symptomatic and asymptomatic SARS-CoV-2 infected individuals	15
Table 9	Observed within-household final size distribution in Vo’	15
Table 10	Parameter estimates from the within-household transmission model	16
Table 11	Parameter estimates from the transmission model fitted to the prevalence data among traced contacts and in the study population	17
Table 12	Real-time RT–PCR primers and probes	17

Supplementary References

17

Supplementary Methods

Section 1. Serological test details

Abbott

ARCHITECT SARS-CoV-2 IgG assay is a chemiluminescent microparticle immunoassay (CMIA) proposed for the qualitative detection of IgG class antibodies against N recombinant SARS-CoV-2 antigen. The test was performed on ARCHITECT i2000SR platform as indicated by the manufacturer. The serum (150 µL), the SARS-CoV-2 antigen coated paramagnetic microparticles and the assay diluent were mixed and underwent a first incubation. If IgG antibodies against N SARS-CoV-2 antigen were present in the sample, they bound to the SARS-CoV-2 antigen coated microparticles. Following a washing cycle, anti-human IgG acridinium-labelled conjugate was added and the reaction mixture was incubated again. After a second washing, the reagent solutions were added and the resulting chemiluminescent reaction was measured as a relative light unit (RLU) and then expressed in the calculated Index (S/C). The cut off is 1.4 Index (S/C), every result <1.4 was considered negative and any result >=1.4 positive.

DiaSorin

LIAISON® SARS-CoV-2 S1/S2 IgG produced by DiaSorin, is a chemiluminescence immunoassay (CLIA) test. It is employed for the quantitative measurement of antibodies of class IgG directed against antigens S1 and S2 of SARS-CoV-2 and its results have been directly related to the titres of neutralizing antibodies against SARS-CoV-2 identified by plaque-reduction neutralization test (PRNT) [1]. The test was performed on LIAISON® XL Analyzer as indicated by the manufacturer.
In brief, magnetic particles coated by recombinant S1 and S2 specific antigens were used as solid phase in presence of mouse monoclonal antibodies anti-human IgG bound to an isoluminol derivative.
During the first incubation, the anti-SARS-CoV-2 IgG antibodies sited in the calibrators, controls and possibly samples, bound to the solid phase via recombinant S1 and S2 antigens. During the second incubation, the conjugated mouse antibodies reacted with the anti-SARS-CoV-2 IgG already bound to the solid phase. After each incubation, unbound material was removed by a wash cycle. The starter reagents were then added to induce a chemiluminescence reaction. The light signal, and then the quantity of antibody-isoluminol conjugate still present as bound to the IgG, was measured by a photomultiplier, in relative light units (RLU), then expressed in arbitrary units (AU/mL). A result < 12 AU/ml has to be evaluated as negative, from 12 to 15 AU/ml equivocal and >15 AU/ml positive.

Roche

Elecsys® Anti-SARS-CoV-2 is an electro-chemiluminescence immunoassay (ECLIA) based on double-antigen sandwich assay, intended for the qualitative detection of IgG antibodies directed against SARS-CoV-2 N antigen in human serum and plasma. The test was performed on Cobas e 601 Analyzer as indicated by the manufacturer.
In sum, 20 µL of the patient's serum were incubated in presence of biotinylated and ruthenylated N antigen. If SARS-CoV-2 antibodies were in the sample, they developed double-antigen sandwich immune complexes with the recombinant N antigen. During the second incubation, the addition of streptavidin-coated microparticles allowed the binding of the complexes to the solid phase. The particles composing the solid phase were then magnetically captured onto the surface of an electrode to which a voltage was applied, inducing an electrochemiluminescence reaction. The signal was measured with a photomultiplier and expressed as an index. Therefore, a result < 1 cut-off index (COI) was evaluated as negative, >=1 COI as positive.

Section 2. Contact and ground truth definitions

Contact definition

'Direct contact' definition includes i) contacts as reported by the infected subjects in the contact tracing forms or in the follow up interviews (denoted with code "1" in the dataset) and ii) contacts inferred based on household composition (denoted with code "3"). The 'indirect contacts' definition includes subjects who had a direct contact with a subject having a positive Abbott, DiaSorin, Roche or PCR test (i.e., positive to either test, denoted with code "2" in the dataset). Due to the limited information on the type of contact occurring in two contact settings (variables ending in 'place' in the data), we considered all contacts reported in those settings as indirect contacts.

Ground truth definitions

In the baseline definition, we considered as SARS-CoV-2 infected all subjects who had (a) positive PCR test in February or March 2020 and/or (b) positive results to two serological tests with different target and/or (c) micro-neutralisation titres > 1:40 (1/dil). The 'direct contacts' definition defines as SARS-CoV-2 infections all subjects who met the baseline definition and also includes subjects who had a positive result to any serological assay and a history of direct contact with an infection meeting the baseline ground truth definition. The 'indirect contacts' definition defines as SARS-CoV-2 infection all subjects who met the criteria of the 'direct contact' definition plus those who had a positive result to any serological assay and a history of indirect contact with a subject meeting the baseline or direct contact ground truth definition (**Table S1**).

Section 3. Seroprevalence estimates details

The log-likelihood of the model is given by:

$$\begin{aligned} ll(data|\theta, se_A, se_D, se_R, sp_A, sp_D, sp_R) \\ = \sum_{i\in(A,D,R)} P_i^{se}\ln(se_i) + (T_i^{se} - P_i^{se})\ln(1-se_i) \\ + P_i^{sp}\ln(sp_i) + (T_i^{sp} - P_i^{sp})\ln(1-sp_i) \\ + \sum_{j\in(+,-)} X_{A_jD_jR_j}\, ln(P(A_jD_jR_j)) \end{aligned} \quad (1)$$

where T_i^{se} and T_i^{sp} respectively denote the number of samples tested in-house to assess the sensitivity and specificity of assay i (**Table 3**, see footnote); P_i^{se} and P_i^{sp} respectively denote the number of positive samples from the in-house experiments assessing the sensitivity and specificity of assay i (**Table 3**, see footnote); se_i and sp_i denote the sensitivity and specificity parameters of assay i; and $X_{A_jD_jR_j}$ denotes the number of samples in category $A_jD_jR_j$ for j positive (+) or negative (-) (**Table S2**, May). Let θ denote the probability of having been infected by SARS-CoV-2. The probability of observing $A_jD_jR_j$ is given by

$$P(A_jD_jR_j) = P(A_jD_jR_j|\, infected)\,\theta + P(A_jD_jR_j|\, not\ infected)(1-\theta) \quad (2)$$

where

$$P(A_jD_jR_j|\, infected) = \prod_{T\in(A,D,R)} (1_{T_+}(T_j)(se_T) + 1_{T_-}(T_j)(1-se_T)) \quad (3)$$

$$P(A_jD_jR_j|\, not\ infected) = \prod_{T\in(A,D,R)} (1_{T_+}(T_j)(1-sp_T) + 1_{T_-}(T_j)(sp_T)) \quad (4)$$

and $1_{T_+}(T_j) = 1$ if $j = +$ and 0 otherwise, while $1_{T_-}(T_j) = 1$ if $j = -$ and 0 otherwise. We assumed uniform prior distributions and explored the posterior distribution of the parameters using the Metropolis-Hastings algorithm using 100,000 iterations, a thinning factor of 1 every 100 and a burn-in period of 100 samples. The p-value was calculated from the goodness of fit chi-squared statistic with 6 degrees of freedom, obtained from the central posterior estimates. For each assay, we estimate the

positive predictive value (PPV) and negative predictive value (NPV) from the posterior distribution of the assay-specific sensitivity, specificity and prevalence using equations (5) and (6)

$$PPV_T = \frac{se_T\,\theta}{se_T\,\theta + (1 - sp_T)(1 - \theta)} \quad (5)$$

$$NPV_T = \frac{sp_T\,(1-\theta)}{sp_T\,(1-\theta) + (1 - se_T)\theta} \quad (6)$$

Section 4. Within-household transmission model

We quantified the extent of within-household SARS-CoV-2 transmission implementing the methods developed by Fraser et al.[2], which are an extension of the classic Reed-Frost chain-binomial model. Let $k_{(m,n)}$ denote the number of households of size n with m infections. The mean attack rate by household size is the proportion of subjects infected by household size and is defined as

$$AR(n) = \frac{\sum_{m=0}^{n} m\, k_{(m,n)}}{n \sum_{m=0}^{n} k_{(m,n)}} \quad (7)$$

The secondary attack rate by household size is the proportion of infected household members of an infected subject and is defined as

$$SAR(n) = \frac{\sum_{m=1}^{n} (m-1)\, k_{(m,n)}}{(n-1) \sum_{m=1}^{n} k_{(m,n)}} \quad (8)$$

The number of non-primary infections by household size is given by $(n-1)SAR(n)$.
The household attack rate by household size is the proportion of households with at least one infected household member and is defined as

$$HAR(n) = \frac{\sum_{m=1}^{n} k_{(m,n)}}{\sum_{m=0}^{n} k_{(m,n)}} \quad (9)$$

The household size distribution is denoted

$$pr[n] = \frac{1}{N_H \sum_{m=0}^{n} k_{(m,n)}} \quad (10)$$

where N_H is the number of households sampled $N_H = \sum_{n=1}^{n_{max}} \sum_{m=0}^{n} k_{(m,n)}$. Let F_m^{n,s_0} denote the probability of observing m infections in a household of size n given s_0 susceptible individuals, Q denote the escape probability from sources of infection outside the household (i.e., $1-Q$ denotes the probability of infection from sources outside the household) and h_n the hazard of infection for each members of a household of size n. Fraser et al.[2] show that F_m^{n,s_0} can be estimated as the solution of the system of equations

$$\binom{s_0}{k} = \frac{\sum_{m=0}^{k} \binom{s_0 - m}{k - m} F_m^{n,s_0}}{\Phi_n(s_0 - k)^m Q^{(s_0-k)}} \qquad for\ \ k = 0, \dots, s_0 \quad (11)$$

where $\Phi_n(x) = \mathbb{E}[\exp(-h_n x)]$ is the moment generating function of the distribution of hazards in households of size n. The Susceptible-Infectious Transmission Probability (SITP) is given by $SITP_n = 1 - \Phi_n(1)$. Following Fraser et al.[2], the baseline assumption is that $\Phi_n(x) = q^x = e^{-\beta x}$. In model variants V we assume that h_n has a Gamma distribution with mean B_n and shape k, i.e., $\Phi_n(x) = k/(k + xB_n)^k$. In model variant P we assume that $B_n = \beta/n^\alpha$ and $B_n = \beta$ otherwise. In model variant X we assume that a proportion p_i of subjects isolate and the probability of observing m infections in a household of size n with s_0 susceptible individuals is given by

$$S_m^{n,s_0} = \sum_{r=0}^{s_0 - m} Bin(r, p_i, n)\, F_m^{n,s_0 - r} \quad (12)$$

In model variant A we allow for a proportion of subjects p_{srev} to serorevert (i.e., test seronegative despite having been infected) and in this model variant the probability of observing m infections in a household of size n with s_0 susceptible individuals is given by

$$T_m^{n,s_0} = \sum_{t=0}^{s_0-m} Bin(r, p_{srev}, m+t)\, S_{m+t}^{n,s_0} \tag{13}$$

All models estimate Q and β and the basic Reed-Frost model is obtained for $\alpha = 0$, $k \longrightarrow +\infty$, $p_i = 0$ and $p_{srev} = 0$ which are the default parameter values. We explore all possible 2^4 model variants (i.e., parameter combinations). For instance, model PVX estimates Q, β, α, k and p_i.

We define model variant Z, an extension of model variant P, with an additional parameter z that provides an alternative interpolation between frequency and density dependent transmission, $B_n = \beta/(n-z)^\alpha$. We also tested a two-group model, denoted by T, where the moment generating function was given by

$$\phi_{2-group}(x) = p_{high} e^{-\frac{\beta_{high}}{n^\alpha}} + (1 - p_{high}) e^{-\frac{\beta_{low}}{n^\alpha}} \tag{14}$$

The final distribution was given by $P_m^n = T_m^{n,n}$ and parameter inference was conducted in a Bayesian framework, using the Metropolis-Hastings algorithm. We used the Deviance Information Criterion (DIC) for model selection, which is based on the deviance

$$Dev = 2 \sum_{m,n\ s.t. k_{(m,n)}>0} k_{(m,n)} \left(\ln(O_m^n) - (\ln (P_m^n)\right) \tag{15}$$

where $O_m^n = k_{(m,n)} / \sum_{j=0}^{n} k_{(j,n)}$. We used uniform prior distributions, run the chains for 10,000 iterations, thinned them by a factor of 1/100 and used a burn-in period of 100 iterations.

The overall Susceptible Infectious Transmission Probability (SITP) was estimated from 1,000 samples of the posterior distribution using formula

$$SITP = 1 - \sum_{n=0}^{n_{max}} \phi_n(1) f[n] \tag{16}$$

where $f[n] = n\, pr[n]/\mathbb{E}[n]$ and $\mathbb{E}[n]$ denotes the mean household size $\mathbb{E}[n] = \sum_{n=1}^{n_{max}} n\, pr[n]$. Full details are given in Fraser et al.[2].

Following Lloyd-Smith et al.[3], we model the expected proportion of transmission due to infectious individuals with reproduction number $\nu < x$ as

$$F_{trans}(x) = \frac{1}{R_0} \int_0^x u f_\nu(u) du \tag{17}$$

where $f_\nu(x)$ is the probability density function of the individual reproduction number, which was assumed to be gamma distributed. Fraser et al.[2] demonstrate that parameter k in the moment generating function of the distribution of hazards in households of size n is equivalent to the shape parameter of the individual reproduction number distribution $f_\nu(x)$ [3]. The expected proportion of transmission due to individuals with $\nu > x$ is $1 - F_{trans}(x)$.The proportion of individuals with $\nu > x$ is $1 - F_\nu(x)$, where $F_\nu(x)$ is the cumulative density function of the individual reproduction number distribution. Panel c of **Figure 5** shows $1 - F_{trans}(x)$ on the y-axis versus $1 - F_\nu(x)$ on the x-axis, having used $R_0 = 2.4$.

Section 5. SARS-CoV-2 transmission model with contact tracing

Dynamics of SARS-CoV-2 transmission without contact tracing

The flow diagram of the transmission model is given in **Supplementary Figure S4**. Following Lavezzo et al.[22], we assumed that the population of Vo' was fully susceptible to SARS-CoV-2 (S compartment) at the start of the epidemic. Upon infection, subjects incubate the virus (E compartment) and have undetectable viraemia for an average of 1/ν days before entering a stage (TP^{pre} compartment) that lasts an average of 1/δ days, in which subjects show no symptoms and have detectable viraemia. We assume that a proportion p of the infected population remains asymptomatic throughout the whole course of the infection (I_A compartment) and that the remaining proportion $1 - p$ develops symptoms (I_S compartment). We assume that symptomatic (I_S), asymptomatic (I_A+pTP^{pre}) and pre-symptomatic ((1-p)TP^{pre}) subjects contribute to the onward transmission of SARS-CoV-2 and that symptomatic, asymptomatic and pre-symptomatic subjects transmit the virus for an average of 1/δ +

1/γ days. We further assume that the virus can be detected by swab testing beyond the duration of the infectious period; this assumption is compatible with the hypothesis that transmission occurs for viral loads above a certain threshold but the diagnostic test can detect the presence of virus below the threshold for transmission. Compartments TP^{post}_S and TP^{post}_A respectively represent symptomatic and asymptomatic subjects who are no longer infectious but have a detectable viral load, and hence test positive. Eventually, the viral load of all infections decreases below detection and subjects move into a test negative (TN) compartment. We assume a step change in the reproduction number on the day that lockdown started. We assume that the reproduction number is given by $R_0^1 = \beta(\frac{1}{\gamma} + \frac{1}{\delta})$ at the start of the epidemic and that it drops to $R^2 = w\, R_0^1$ after the start of the lockdown, where $1 - w$ represents the percent reduction in R_0^1 due to the intervention. We let T_i denote the number of subjects swabbed on survey i ($i = 1{,}2$) and let P_{Ai}, P_{Pi} and P_{Si} respectively denote the number of swabs testing positive among asymptomatic, pre-symptomatic (i.e. those showing no symptoms at the time of testing but developing symptoms afterwards) and symptomatic subjects, respectively. We assume that the number of positive swabs among symptomatic, pre-symptomatic and asymptomatic infections on survey i follows a binomial distribution with parameters T_i and π_{Xi}, where π_{Xi} represents the probability of testing positive on survey i for class X (= A,S). For symptomatic subjects, π_{Si} is given by $\pi_{Si} = \frac{I_S(t_i)+TP_S^{post}(t_i)}{N}$, for asymptomatic subjects π_{Ai} it is given by $\pi_{Ai} = \frac{pTP^{pre}(t_i)+I_A(t_i)+TP_A^{post}(t_i)}{N}$ and for pre-symptomatic subjects π_{Pi} is given by $\pi_{Pi} = \frac{(1-p)TP^{pre}(t_i)}{N}$, assuming perfect diagnostic sensitivity and specificity.

Modelling contact tracing

We modelled the effect of contact tracing by adding compartments indexed by Q into the model (green compartments in **Supplementary Figure S4**), representing susceptible traced subjects in quarantine (S_Q) and infected traced subjects isolated (during any stage of the infection, E_Q, TP^{pre}_Q, I_Q, TP^{post}_Q and TN_Q). We assumed that susceptible subjects were detected and quarantined at rate ct_S and that infected subjects (during any stage of the infection, E_Q, TP^{pre}_Q, I_Q, TP^{post}_Q and TN_Q) were detected and isolated at a rate ct_I. We assumed two differential rates of detection and isolation to capture the simultaneous implementation of contact tracing with mass testing, which contributed to the detection of infected subjects by contact tracing. We assumed complete isolation of traced subjects, i.e., that isolated infected subjects did not transmit the disease onwards and, given the time scale of the epidemic in Vo', that quarantined susceptible subjects were completely protected against the infection for the whole duration of the first wave. We assumed that contact tracing started on 24th February 2020. The probability that traced contacts ever testing positive is given by $p_{ct+} = 1 - \frac{S_Q}{N_Q}$, where $N_Q = S_Q + E_Q + TP_Q^{pre} + I_Q + TP_Q^{post} + TN_Q$. We assumed that the observed cumulative number of PCR positive traced subjects $CT^+(= 44)$ followed a binomial distribution with parameters CT^{traced} $(= 190)$ and probability of ever having tested positive p_{ct+}. The probability of being traced among infected subjects is given by $p_{traced} = \frac{N_Q - S_Q}{N - S + N_Q - S_Q}$. We assumed that the number of PCR positive traced subjects $CT^+(= 44)$ followed a binomial distribution with parameters P $(= 100)$ and probability p_{traced}.

Calibration and parameter inference

The likelihood of the model is given by the product of the binomial distributions for symptomatic, pre-symptomatic and asymptomatic subjects at times t_i, $i = 1{,}2$, the probability that traced contacts test PCR positive and the probability that PCR positive subjects are traced. Inference was conducted in a Bayesian framework, using the Metropolis-Hastings Markov Chain Monte Carlo (MCMC) method with uniform prior distributions. We fixed the average generation time (equal to 1/ν + 1/δ + 1/γ) to 7 days and let the model infer 1/ν and 1/δ. We explored the following values of

R_0^1: 2.1, 2.4, 2.7, which are compatible with a doubling time of 3-4 days, as observed in Vo' and elsewhere in the Veneto region. We assumed that seeding of the infection occurred on 4 February 2020. Following the results obtained in Lavezzo et al.[4], we assumed a fixed average duration of viral detectability beyond the infectious period $1/\sigma$ equal to 4 days. We estimate the number of infections introduced in the population from elsewhere at time t_0 (4 February 2020), the proportion of asymptomatic infections p, the average durations $1/\nu$, $1/\delta$ and $1/\gamma$, the percent reduction in R_0^1 due to mass testing and the implementation of the lockdown $(1-w)100\%$ and the rates of isolation of susceptible traced contacts ct_S and infected traced contacts ct_I.

Counterfactual analysis

We fitted our baseline scenario including mass testing and lockdown and contact tracing (MT + CT scenario) to the data collected from Vo'. In a counterfactual analysis, we simulated the impact on the epidemic final size of each intervention implemented in isolation, i.e., (i) mass testing and the lockdown in the absence of contact tracing (MT scenario), and (ii) contact tracing in the absence mass testing and the lockdown (CT scenario). For both scenarios, we sampled 100 realisations from the posterior distribution of the parameters and in the MT scenario we simulated from the model having assumed no isolation due to contact tracing ($ct_S = 0$ and $ct_I = 0$); in the CT scenario we simulated from the model having assumed no reduction in the reproduction number due to mass testing and lockdown ($w = 0$). We also simulated what impact increased or reduced contact tracing would have had on the epidemic final size. We explored the following scenarios: (iii) mass testing and lockdown in the presence of contact tracing with reduced (half) the estimated contact tracing efforts (MT + CTx0.5 scenario), (iv) mass testing and lockdown in the presence of contact tracing with enhanced (double) the estimated contact tracing efforts (MT + CTx2 scenario); (v) contact tracing implemented in the absence of mass testing and lockdown with enhanced (double) the estimated contact tracing efforts (CTx2 scenario); (vi) contact tracing implemented in the absence of mass testing and lockdown with enhanced (four times) the estimated tracing efforts (CTx4 scenario). In the scenarios with enhanced or reduced contact tracing efforts we multiplied the rates of isolation due to contact tracing by the assumed multiplier (e.g., for the CTx2 scenario ct_S and ct_I were fixed to 2 times the sampled realisations from the posterior distribution). In the CTx0.5, CTx2 and CTx4 scenarios we assumed no mass testing and lockdown effect (i.e., fixed $w = 0$) while in the MT + CTx0.5 and MT + CTx2 scenarios we sampled w for the posterior distribution and fixed ct_S and ct_I to half and twice the sampled posterior values, respectively. For each scenario, we estimated the relative reduction in the epidemic final size compared to the unmitigated scenario by dividing the estimated final size with interventions by the estimated final size without interventions. The relative reductions were estimated by simulating from the model having sampled 100 estimates from the posterior distribution of the parameters.

Supplementary Note 1

Using the baseline ground truth definition, we found no significant trend in the antibody titres with the number of days since symptom onset, and no significant differences in the mean antibody titres of symptomatic versus asymptomatic infections, of hospitalised versus non-hospitalised infections (except for the DiaSorin assay in November, p = 0.04), or by sex (**Supplementary Table S5**).
We found no statistically significant difference in the antibody decay rates of symptomatic versus asymptomatic subjects, nor in hospitalized versus non-hospitalized infections, or by sex (except for DiaSorin, p = 0.05; **Supplementary Table S6)**. Among asymptomatic infections, we observed no significant association between antibody decay rate and BMI (**Supplementary Table S6**). No significant association was found between symptom occurrence and age, nor between symptom occurrence and BMI category, whether or not age groups were included in the model. We also found no significant association between symptoms occurrence and comorbidities (**Supplementary Table S7**) nor between symptoms occurrence and medical treatment (**Supplementary Table S8**).

Supplementary Figures

a

	Abbott	Roche	Neutralisation	DiaSorin
Abbott	1	0.81**	0.48**	0.62**
Roche		1	0.31*	0.43**
Neutralisation			1	0.65**

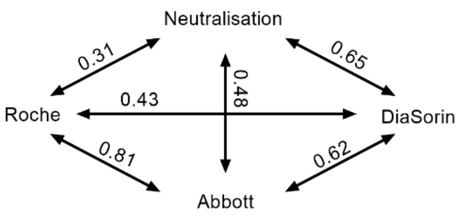

b

	Abbott	Roche	Neutralisation	DiaSorin
Abbott	1	0.77**	0.15	0.37**
Roche		1	-0.09	-0.10
Neutralisation			1	0.50**

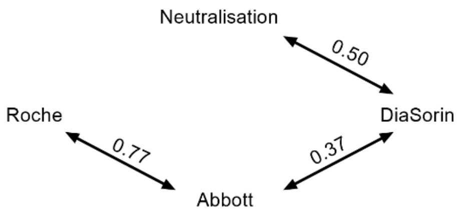

c

	Abbott	Roche	Neutralisation	DiaSorin
Abbott	1	0.89**	0.63**	0.66**
Roche		1	0.54**	0.62**
Neutralisation			1	0.85**

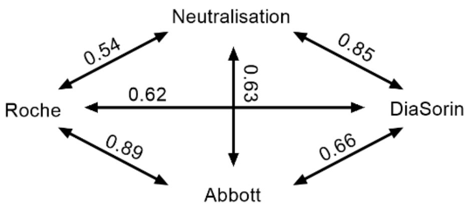

d

	Abbott	Roche	Neutralisation	DiaSorin
Abbott	1	0.82**	0.2	0.06
Roche		1	-0.14	0.15
Neutralisation			1	0.75**

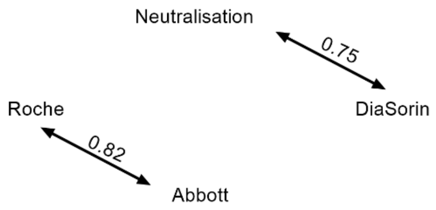

Supplementary figure 1: Association among serological and micro-neutralisation assays. Marginal (a and c) and partial (b and d) Pearson correlations among DiaSorin, Abbott, Roche and micro-neutralisation titres calculated on exposed subjects identified through the baseline ground truth definition in the May (a and b) and November (c and d) serosurveys.

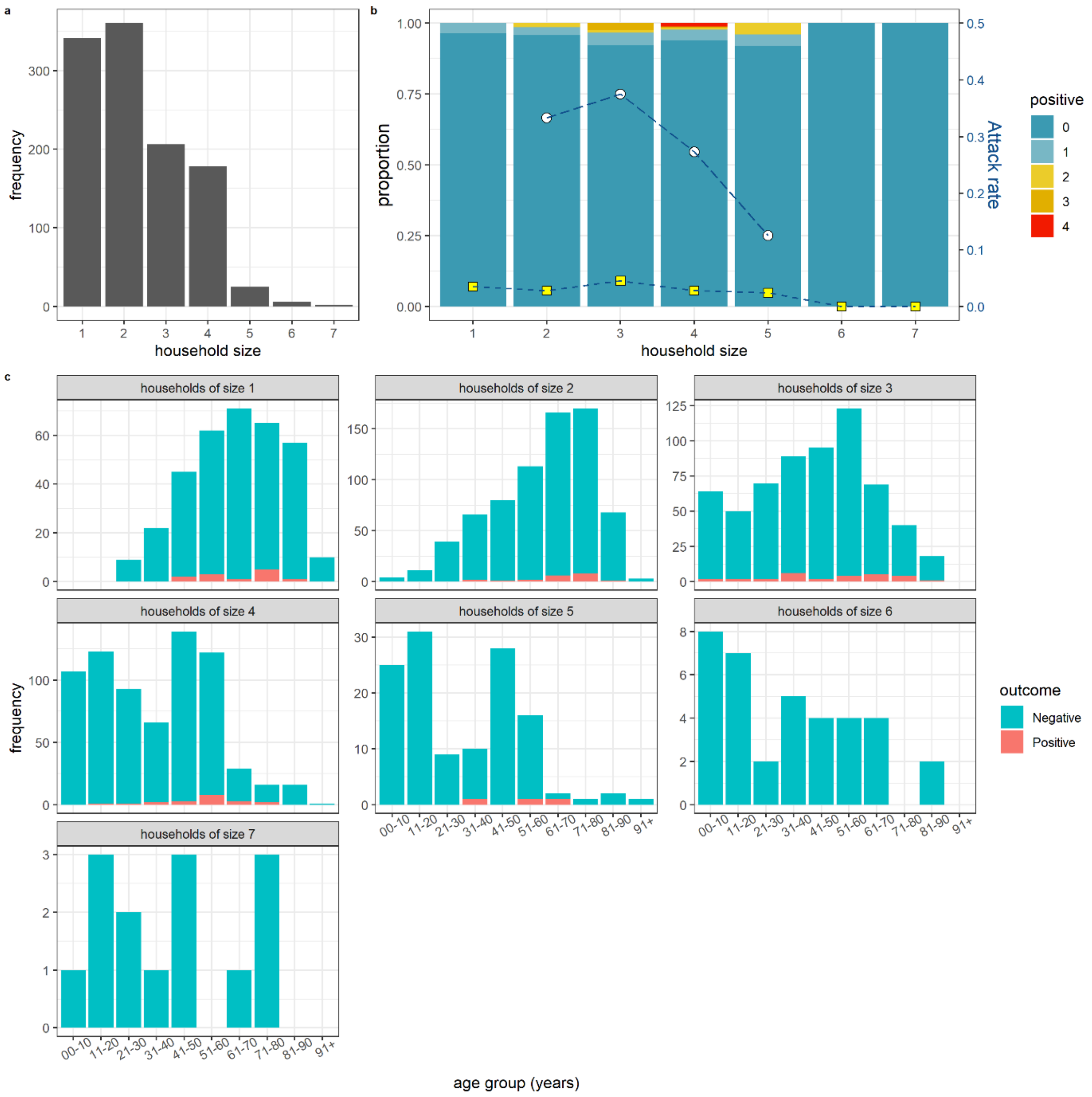

Supplementary figure 2: Household size distributions, number of infections and attack rates by household size. (a) Household size distribution. (b) Proportion of households with 0, 1, 2, 3, and 4 infected household members according to the baseline ground truth definition, by household size. (c) Number of SARS-CoV-2 infected (red) and uninfected (blue) household members by age-group and household size according to the baseline ground truth definition.

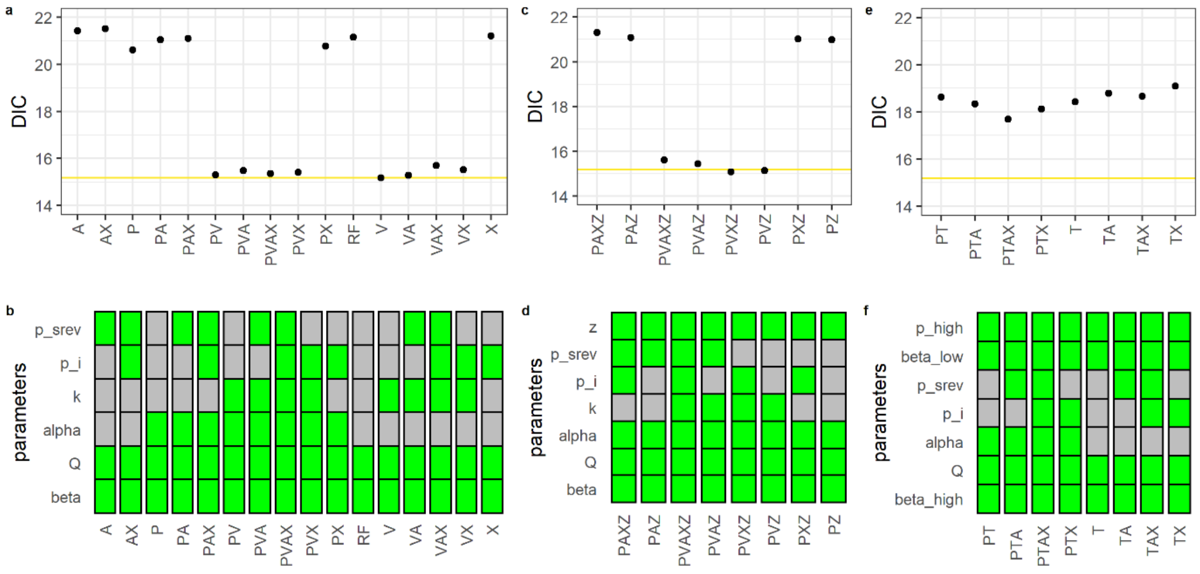

Supplementary figure 3: Within-household transmission models performance metric. (a)-(b) DIC score and estimated parameters by model variant, using the original model proposed by Fraser et al.[2]. (c)-(d) DIC score and estimated parameters by model variant, using the extended model. (e)-(f) DIC score and estimated parameters by model variant, using the two-groups model.

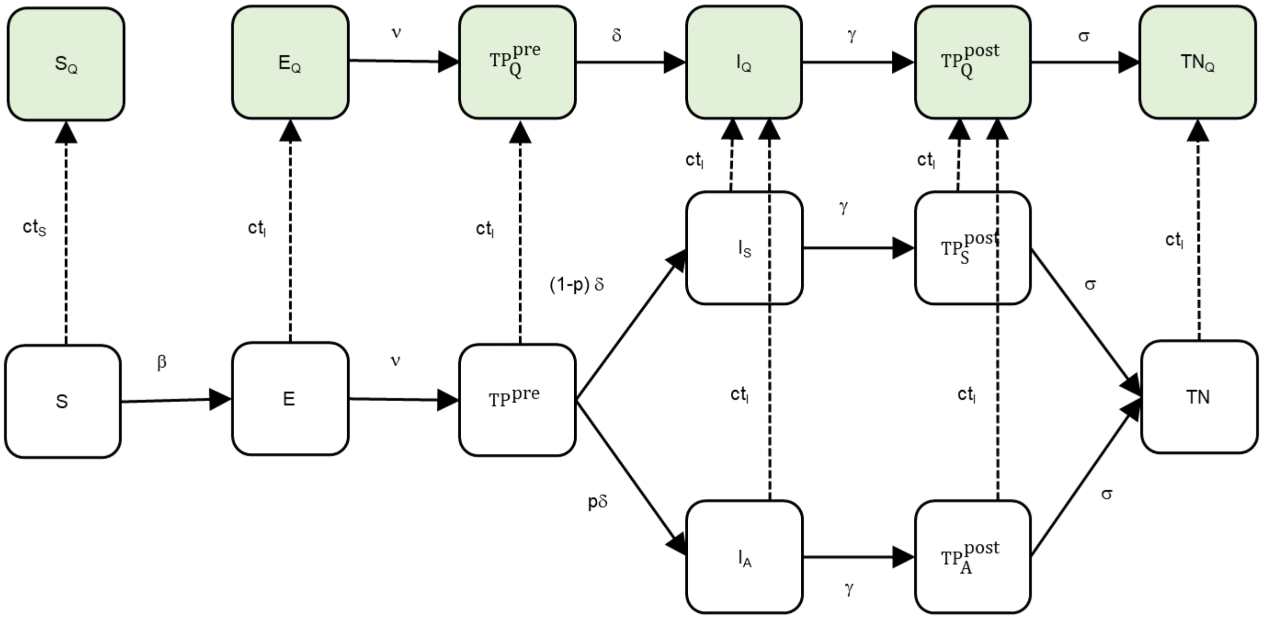

Supplementary figure 4: Flow diagram of the transmission model used to estimate the impact of contact tracing. Shaded compartments represent the new compartment added to the model developed in Lavezzo et al.[2]. Quarantine and isolation are modelled by removing infections from the general community at rates ct_S and ct_I respectively, starting from 24th February 2020 onwards. We assumed a closed population (i.e., no births and deaths) and neglected SARS-CoV-2 mortality due to the small number of COVID-19 deaths (= 3) observed in Vo' during the study period.

Supplementary Tables

Supplementary table 1. SARS-CoV-2 exposure authentication criteria. Criteria of decreasing stringency for the definition of the ground truths (GTs) identifying all individuals exposed to SARS-CoV-2 from the putative start of infection (early February) to the first serosurvey (1-3/05/2020).

Baseline ground truth definition (n = 125)	Direct contact ground truth definition (n = 147)	Indirect contact ground truth definition (n = 161)
Subjects satisfying at least one of the following criteria:	Subjects satisfying at least one of the following criteria:	Subjects satisfying at least one of the following criteria:
♦ Positive swab	♦ Positive swab	♦ Positive swab
♦ Neutralisation >1:40	♦ Neutralisation >1:40	♦ Neutralisation >1:40
♦ Positivity to two serological tests with different antigen target	♦ Positivity to two serological tests with different antigen target	♦ Positivity to two serological tests with different antigen target
	♦ Direct contact with positive case (according to baseline ground truth definition) and positivity to at least one serological test	♦ Direct contact with positive case (according to baseline ground truth definition) and positivity to at least one serological test
		♦ Indirect contact with positive case (according to baseline and direct contact ground truth definition) and positivity to at least one serological test

Supplementary table 2. Observed test results combinations in the May and November 2020 serosurveys. Test results among Vo' residents, including for PCR positive (+) and PCR negative (-) subjects in the February and March 2020 surveys. Equivocal DiaSorin results were not included.

	Vo' residents			
	PCR- Feb/Mar* n = 2,066 (%)	PCR+ Feb/Mar* n = 62 (%)	May n = 2,226 (%)	Nov n = 133 (%)
A+D+R+	14 (0.7)	53 (85.5)	68 (3.1)	29 (21.8)
A+D-R+	2 (0.1)	7 (11.3)	9 (0.4)	1 (0.8)
A+D+R-	0 (0.0)	0 (0.0)	0 (0.0)	0 (0.0)
A-D+R+	0 (0.0)	0 (0.0)	0 (0.0)	40 (30.1)
A+D-R-	6 (0.3)	0 (0.0)	6 (0.3)	4 (3.0)
A-D+R-	38 (1.8)	0 (0.0)	38 (1.7)	21 (15.8)
A-D-R+	3 (0.1)	2 (3.2)	5 (0.2)	16 (12.0)
A-D-R-	2,003 (97.0)	0 (0.0)	2,100 (94.3)	22 (16.5)

A+ = Abbott seropositive; A- = Abbott seronegative; D+ = DiaSorin seropositive; D- = DiaSorin seronegative; R+ = Roche seropositive; R- = Roche seronegative. PCR + = PCR positive in either survey conducted in February and March 2020[21]. PCR- = PCR negative in both surveys conducted in February and March 2020[21]. *According to the results observed in the May 2020 serosurvey.

Supplementary table 3. Assay-specific performance against the different ground truth definitions. Sensitivity, specificity, positive predictive value and negative predictive value (mean and 95%CI) of the different assays against the different ground truth definitions.

		Vo cluster (full dataset)			Vo residents		
	Ground truth definition	Abbott	DiaSorin	Roche	Abbott	DiaSorin	Roche
sens	baseline	0.935 (0.863 – 0.976)	0.876 (0.794 – 0.934)	1.000 (0.961 – 1.000)	0.950 (0.877 – 0.986)	0.890 (0.802 – 0.949)	1.000 (0.955 – 1.000)
spec	baseline	0.996 (0.993 – 0.998)	0.980 (0.974 – 0.985)	0.997 (0.994 – 0.999)	0.996 (0.992 – 0.998)	0.980 (0.974 – 0.986)	0.997 (0.994 – 0.999)
PPV	baseline	0.905 (0.846 – 0.962)	0.630 (0.551 – 0.714)	0.939 (0.882 – 0.981)	0.894 (0.827 – 0.953)	0.619 (0.523 – 0.705)	0.930 (0.869 – 0.978)
NPV	baseline	0.997 (0.995 – 0.999)	0.995 (0.992 – 0.998)	1.000 (1.000 – 1.000)	0.998 (0.996 – 1.000)	0.996 (0.993 – 0.998)	1.000 (1.000 – 1.000)
sens	direct contacts	0.802 (0.715 – 0.871)	0.881 (0.809 – 0.934)	0.847 (0.766 – 0.908)	0.798 (0.705 – 0.872)	0.893 (0.817 – 0.945)	0.828 (0.739 – 0.897)
spec	direct contacts	0.997 (0.994 – 0.999)	0.987 (0.982 – 0.991)	0.998 (0.996 – 1.000)	0.997 (0.994 – 0.999)	0.988 (0.983 – 0.992)	0.998 (0.995 – 0.999)
PPV	direct contacts	0.937 (0.882 – 0.980)	0.770 (0.697 – 0.838)	0.959 (0.918 – 0.991)	0.929 (0.868 – 0.977)	0.780 (0.704 – 0.855)	0.953 (0.901 – 0.989)
NPV	direct contacts	0.991 (0.987 – 0.994)	0.994 (0.991 – 0.997)	0.993 (0.989 – 0.996)	0.991 (0.987 – 0.994)	0.995 (0.992 – 0.998)	0.992 (0.988 – 0.995)
sens	indirect contacts	0.726 (0.638 – 0.802)	0.886 (0.820 – 0.935)	0.758 (0.673 – 0.830)	0.714 (0.621 – 0.796)	0.897 (0.828 – 0.946)	0.732 (0.640 – 0.811)
spec	indirect contacts	0.998 (0.995 – 0.999)	0.993 (0.988 – 0.996)	0.998 (0.996 – 1.000)	0.998 (0.995 – 0.999)	0.994 (0.990 – 0.997)	0.998 (0.995 – 0.999)
PPV	indirect contacts	0.947 (0.897 – 0.989)	0.867 (0.806 – 0.921)	0.959 (0.918 – 0.991)	0.941 (0.890 – 0.987)	0.890 (0.832 – 0.943)	0.953 (0.907 – 0.991)
NPV	indirect contacts	0.986 (0.981 – 0.990)	0.994 (0.991 – 0.997)	0.987 (0.983 – 0.992)	0.985 (0.980 – 0.990)	0.995 (0.992 – 0.997)	0.986 (0.981 – 0.990)

Sens = sensitivity; spec = specificity; PPV = positive predictive value; NPV = negative predictive value. For sensitivity and specificity, the 95% CI represents the exact binomial CI; for PPV and NPV, the 95% CI has been calculated by bootstrapping.

Supplementary table 4. Positive (+) Serological results on the subjects tested with all three assays in May and November 2020.

	n = 2,443 tested in May*			n = 148 tested in November*		
	DiaSorin+	Roche+	Abbott+	DiaSorin+	Roche+	Abbott+
DiaSorin+	119	77	76	102	79	33
Roche+		94	87		96	34
Abbott+			93			38

*Subjects with equivocal DiaSorin results have been excluded

Supplementary table 5. Association analysis on antibody titres. P-values and tests performed. Significant p-values are in bold. The test used is specified in the footnote. The analysis was performed on the subjects identified as positive using the baseline ground truth definition.

		May			November		
outcome	predictor	Abbott	DiaSorin	Roche	Abbott	DiaSorin	Roche
Antibody titres	Days since symptom onset	0.675[1]	0.760[1]	0.449[1]	0.329[1]	0.774[1]	0.482[1]
Antibody titres	Symptomatic	0.707[2]	0.812[3]	0.614[3]	0.746[3]	0.612[3]	0.926[3]
Antibody titres	Hospitalised	0.062[3]	0.632[3]	0.693[3]	0.433[3]	**0.041[3]**	0.188[3]
Antibody titres	Sex	0.450[2]	0.116[3]	0.262[3]	0.244[3]	0.474[3]	0.132[3]
Antibody titres	Age group	**0.005[4]**	**0.007[4]**	**0.006[4]**	0.174[4]	**0.005[4]**	**0.001[4]**
Antibody titres	BMI category	**0.042[5]**	**0.020[4]**	0.304[4]	0.631[4]	0.129[4]	0.367[4]
Antibody titres among symptomatic	BMI	**0.006[1]**	**0.038[1]**	**0.025[1]**	0.055[1]	0.336[1]	**0.030[1]**
Antibody titres among asymptomatic	BMI	0.834[1]	0.664[1]	0.940[1]	0.840[1]	0.763[1]	0.949[1]

[1] linear regression; [2] t-test; [3] Wilcoxon ranked-sum test; [4] Kruskal-Wallis test; [5]Anova test with equal variances. Two-sided p-values. P-values < 0.05 are in bold. No adjustment for multiple comparisons.

Supplementary table 6. Association analysis on antibody decay rates. P-values and tests performed. Significant p-values are in bold. The test used is specified in the footnote. The analysis was performed on the subjects identified as positive using the baseline ground truth definition.

outcome	predictor	Abbott	DiaSorin	Roche
Antibody decay rate	Symptomatic	0.320[2]	0.120[2]	0.949[2]
Antibody decay rate	Hospitalised	0.429[2]	0.784[2]	0.377[2]
Antibody decay rate	Sex	0.422[2]	**0.047[3]**	0.440[2]
Antibody decay rate	Age group	**0.009[4]**	0.408[5]	**0.0009[4]**
Antibody decay rate	BMI categories	0.782[5]	0.602[5]	0.478[4]
Antibody decay rate among symptomatic infections	BMI	0.192[1]	0.880[1]	**0.012[1]**
Antibody decay rate among asymptomatic infections	BMI	0.938[1]	0.726[1]	0.735[1]

[1] linear regression; [2] t-test; [3] Wilcoxon ranked-sum test; [4] Kruskal-Wallis test; [5]Anova test with equal variances. Two-sided p-values. P-values < 0.05 are in bold. No adjustment for multiple comparisons

Supplementary table 7. Frequency of comorbidities in symptomatic and asymptomatic SARS-CoV-2 infected individuals*

	Ground truth					Ground truth direct contacts					Ground truth indirect contacts				
	symptomatic		asymptomatic			symptomatic		asymptomatic			symptomatic		asymptomatic		
Comorbidity*^	pres.	abs.	pres.	abs.	p	pres.	abs.	pres.	abs.	p	pres.	abs.	pres.	abs.	p
Diabetes	1	81	6	37	0.01	3	86	6	52	0.16	3	88	6	64	0.18
Hypertension	21	61	11	32	1.00	24	65	15	43	1.00	24	67	18	52	1.00
Cardiological disease	3	79	4	39	0.23	4	85	5	53	0.32	4	87	5	65	0.50
Allergies	13	69	8	35	0.80	14	75	10	48	0.82	14	77	15	55	0.41
Respiratory disease	6	76	2	41	0.71	6	83	3	55	1.00	6	85	3	67	0.73
Cancers	1	81	0	43	1.00	1	88	0	58	1.00	1	90	0	70	1.00
Autoimmune disease	2	80	1	42	1.00	2	87	2	56	0.651	2	89	2	68	1.00
Kidney disease	0	82	0	43	-	0	89	0	58	-	0	91	0	70	-

p-value (two-sided) obtained from Fisher's exact test for proportions.
*comorbidity history was collected in 82 (ground truth), 89 (ground truth direct contacts) and 91 (ground truth indirect contacts) symptomatic subjects and 43 (ground truth), 58 (ground truth direct contacts) and 70 (ground truth indirect contacts) asymptomatic subjects
^Some individuals had more than one condition

Supplementary table 8. Frequency of medication type in symptomatic and asymptomatic SARS-Cov-2 infected individuals*

	Ground truth					Ground truth direct contacts					Ground truth indirect contacts				
	symptomatic		asymptomatic			symptomatic		asymptomatic			symptomatic		asymptomatic		
Medication*	pres.	abs.	pres.	abs.	p	pres.	abs.	pres.	abs.	p	pres.	abs.	pres.	abs.	p
Ace inhibitors	6	76	2	41	0.71	6	83	3	55	1.00	6	85	3	67	0.73
ARBs§	6	76	3	40	1.00	8	81	3	55	0.53	8	83	3	67	0.35
Non-steroideal anti-inflammatory drugs (NSAIDs)	1	81	2	41	0.27	1	88	2	56	0.56	1	90	3	67	0.32
Antihypertensive	16	66	11	32	0.49	18	71	15	43	0.43	18	73	18	52	0.45
Diuretics	6	76	0	43	0.09	6	83	0	58	0.08	6	85	1	69	0.14
Anticoagulants	2	80	2	41	0.61	2	87	4	54	0.21	2	89	4	66	0.41
Antiplatelet	0	82	1	42	0.34	0	89	2	56	0.15	0	91	2	68	0.19
Hypoglycemic	1	81	4	39	0.05	2	87	4	54	0.22	2	89	4	66	0.41
Thyroid hormones	4	78	3	40	0.69	5	84	4	54	0.74	5	86	4	66	0.51
Immunosuppressants	2	80	1	42	1.00	2	87	1	57	1.00	2	89	1	69	1.00
Corticosteroids	4	78	2	41	1.00	4	85	3	55	1.00	4	87	3	67	1.00
PPI±	4	78	3	40	0.69	6	83	4	54	1.00	7	84	4	66	0.76
Statins	8	74	9	34	0.10	10	79	12	46	0.16	10	81	12	58	0.36
Allopurinol	0	82	1	42	0.34	1	88	1	57	1.00	1	90	1	69	1.00

p-value (two-sided) obtained from Fisher's exact test for proportions.
*medication history was collected in in 82 (ground truth), 89 (ground truth direct contacts) and 91 (ground truth indirect contacts) symptomatic subjects and 43 (ground truth), 58 (ground truth direct contacts) and 70 (ground truth indirect contacts) asymptomatic subjects.
§ARBs: Angiotensin II Receptor Blockers
±PPI: Proton pump inhibitors

Supplementary table 9. Observed within-household final size distribution in Vo'. Observed number of infections (rows) by household size (column) using the baseline ground truth definition.

Number of infections	Household size						
	1	2	3	4	5	6	7
0	329	345	190	167	23	6	2
1	12	10	9	7	1	0	0
2	0	5	2	1	1	0	0
3	0	0	5	1	0	0	0
4	0	0	0	2	0	0	0

Supplementary table 10. Parameter estimates from the within-household transmission model. Mean and 95% CrI of the parameter estimated for each model variant using the data shown in Table S9. DIC denotes the Deviance Information Criterion.

Model	beta	Q	alpha	k	p_pi	p_srev	z	beta_low	p_high	DIC
A	0.29	0.98	-	-	-	0.01	-	-	-	21.4
	(0.18 -0.41)	(0.97-0.98)				(0.00-0.07)				
AX	0.30	0.98	-	-	0.03	0.01	-	-	-	21.5
	(0.20-0.46)	(0.97-0.98)			(0.00-0.28)	(0.00-0.08)				
P	0.36	0.98	0.14	-	-	-	-	-	-	20.6
	(0.20-0.79)	(0.97-0.98)	(0.00-0.83)							
PA	0.35	0.98	0.11	-	-	0.01	-	-	-	21.1
	(0.20-0.76)	(0.97-0.98)	(0.00-0.77)			(0.00-0.09)				
PAX	0.39	0.98	0.15	-	0.04	0.01	-	-	-	21.1
	(0.21-0.91)	(0.97-0.98)	(0.00-0.91)		(0.00-0.33)	(0.00-0.09)				
PV	0.86	0.98	0.08	0.32	-	-	-	-	-	15.3
	(0.29-1.89)	(0.97-0.98)	(0.00-0.69)	(0.08-1.10)						
PVA	0.83	0.98	0.08	0.33			-	-	-	15.5
	(0.28-1.86)	(0.97-0.98)	(0.00-0.66)	(0.08-1.18)						
PVAX	0.87	0.98	0.07	0.32	0.02	0.01	-	-	-	15.4
	(0.30-1.86)	(0.97-0.98)	(0.00-0.65)	(0.08-1.08)	(0.00-0.17)	(0.00-0.09)				
PVX	0.82	0.98	0.07	0.34	0.02	-	-	-	-	15.4
	(0.30-1.82)	(0.97-0.98)	(0.00-0.66)	(0.08-1.30)	(0.00-0.15)					
PX	0.40	0.98	0.15	-	0.05	-	-	-	-	20.8
	(0.21-0.98)	(0.97-0.98)	(0.00-0.94)		(0.00-0.40)					
RF	0.29	0.98	-	-	-	-	-	-	-	21.2
	(0.19–0.42)	(0.97–0.98)								
V	0.78	0.98	-	0.33	-	-	-	-	-	15.2
	(0.28-1.84)	(0.97-0.98)		(0.08-1.13)						
VA	0.80	0.98	-	0.32	-	0.01	-	-	-	15.3
	(0.28-1.80)	(0.97-0.98)		(0.08-1.25)		(0.00-0.09)				
VAX	0.86	0.98	-	0.31	0.02	0.01	-	-	-	15.7
	(0.31-1.87)	(0.97-0.98)		(0.07-1.12)	(0.00-0.19)	(0.00-0.09)				
VX	0.82	0.98	-	0.31	0.02	-	-	-	-	15.5
	(0.28-1.88)	(0.97-0.98)		(0.08-1.17)	(0.00-0.18)					
X	0.30	0.97	-	-	0.03	-	-	-	-	21.2
	(0.19-0.46)	(0.97-0.98)			(0.00-0.26)					
PAXZ	0.37	0.98	0.13	-	0.03	0.01	0.08	-	-	21.3
	(0.20-0.85)	(0.97-0.98)	(0.00-0.87)		(0.00-0.22)	(0.00-0.09)	(0.00-0.66)			
PAZ	0.36	0.98	0.15	-	-	0.01	0.08	-	-	21.1
	(0.21-0.93)	(0.97-0.98)	(0.00-0.92)			(0.00-0.08)	(0.00-0.74)			
PVAXZ	0.92	0.98	0.08	0.32	0.02	0.02	0.09	-	-	15.6
	(0.31-1.89)	(0.97-0.98)	(0.00-0.68)	(0.09-1.09)	(0.00-0.13)	(0.00-0.20)	(0.00-0.77)			
PVAZ	0.87	0.98	0.07	0.32	-	0.01	0.08	-	-	15.4
	(0.30-1.86)	(0.97-0.98)	(0.00-0.60)	(0.08-1.05)		(0.00-0.09)	(0.00-0.78)			
PVXZ	0.90	0.97	0.07	0.34	0.02	-	0.09	-	-	15.1
	(0.30-1.86)	(0.97-0.98)	(0.00-0.66)	(0.08-1.42)	(0.00-0.22)		(0.00-0.79)			
PVZ	0.81	0.98	0.08	0.34	-	-	0.09	-	-	15.1
	(0.29-1.76)	(0.97-0.98)	(0.00-0.69)	(0.08-0.70)			(0.00-0.72)			
PXZ	0.38	0.98	0.15	-	0.03	-	0.07	-	-	21.0
	(0.20-0.88)	(0.97-0.98)	(0.00-0.83)		(0.00-0.24)		(0.00-0.60)			
PZ	0.36	0.98	0.14	-	-	-	0.08	-	-	21.0
	(0.19-0.81)	(0.97-0.98)	(0.00-0.86)				(0.00-0.65)			
PT	1.10	0.98	0.08	-	-	-	-	0.09	0.35	18.6
	(0.33-1.92)	(0.97-0.98)	(0.00-0.71)					(0.00-0.43)	(0.00-0.78)	
PTA	1.06	0.98	0.06	-	-	0.08	-	0.06	0.39	18.3
	(0.34-1.89)	(0.97-0.98)	(0.00-0.55)			(0.00-0.63)		(0.00-0.30)	(0.00-0.79)	
PTAX	1.07	0.98	0.06	-	0.01	0.07	-	0.05	0.41	17.7
	(0.38-1.89)	(0.97-0.98)	(0.00-0.53)		(0.00-0.08)	(0.00-0.68)		(0.00-0.29)	(0.00-0.72)	
PTX	1.14	0.98	0.08	-	0.01	-	-	0.05	0.39	18.1
	(0.37-1.96)	(0.97-0.98)	(0.00-0.61)		(0.00-0.07)			(0.00-0.28)	(0.00-0.71)	
T	1.04	0.98	-	-	-	-	-	0.06	0.37	18.4
	(0.33-1.90)	(0.97-0.98)						(0.00-0.29)	(0.01-0.78)	

TA	1.01	0.98	-	-	-	0.07	-	0.06	0.38	18.8
	(0.33-1.86)	(0.97-0.98)				(0.00-0.72)		(0.00-0.29)	(0.00-0.77)	
TAX	1.09	0.98	-	-	0.01	0.09	-	0.06	0.35	18.7
	(0.38-1.90)	(0.97-0.98)			(0.00-0.08)	(0.00-0.77)		(0.00-0.33)	(0.17-0.65)	
TX	1.09	0.98	-	-	0.01	-	-	0.07	0.35	19.1
	(0.32-1.90)	(0.97-0.98)			(0.00-0.09)			(0.00-0.32)	(0.00-0.75)	

Supplementary table 11. Parameter estimates from the transmission model fitted to the prevalence data among traced contacts and in the study population. Parameter estimates obtained from the fit of the dynamical transmission model described in Figure S4 and in Supplementary Methods, section 5 to the observed prevalence of symptomatic, pre-symptomatic and asymptomatic infections in the first and second surveys, to the observed prevalence of infection among traced contacts and to the observed sensitivity of contact tracing using the Metropolis-Hastings algorithm. Mean and 95% CrI of the parameter estimated for each assumed value of R_0^1.

R_0^1	seed	p	1/ν	1/δ	ct_I	ct_S	1/γ	1-w	DIC
**2.1**	4.80	0.41	0.43	1.73	0.02	0.0021	4.84	0.78	48.19
	(2.97, 9.21)	(0.32, 0.50)	(0.01, 2.15)	(0.95, 2.73)	(0.02, 0.03)	(0.0012, 0.0033)	(3.38, 5.78)	(0.61, 0.98)	
**2.4**	3.31	0.41	1.17	1.49	0.02	0.0022	4.34	0.83	47.62
	(1.26, 9.15)	(0.31, 0.50)	(0.01, 4.99)	(0.63, 2.48)	(0.02, 0.03)	(0.0012, 0.0041)	(1.13, 5.78)	(0.65, 1.00)	
**2.7**	3.26	0.41	2.76	1.15	0.02	0.0027	3.08	0.91	48.11
	(1.09, 6.78)	(0.31, 0.50)	(0.44, 5.84)	(0.51, 2.01)	(0.02, 0.03)	(0.0013, 0.0051)	(0.47, 5.40)	(0.73, 1.00)	

R_0^1 *represents the reproduction number before the implementation of lockdown, p represents the proportion of asymptomatic infections,* 1/ν represents the average time from infection to virus detectability, 1/δ represents the average time from virus detectability to symptoms onset, 1/δ + *1/*γ represents the average duration of the infectious period, ct_I and ct_S denote the rate of detection and isolation of infected and susceptible individuals respectively, *and* 1 − *w represents the reduction in transmissibility after the implementation of lockdown on 24 February 2020. DIC denotes the Deviance Information Criterion.*

Supplementary table 12. Real-time RT–PCR primers and probes.

Primer name	Primer sequence	Target gene
E_Sarbeco_F	ACAGGTACGTTAATAGTTAATAGCGT	E gene
E_Sarbeco_P1	FAM-ACACTAGCCATCCTTACTGCGCTTCG-BBQ	
E_Sarbeco_R	ATATTGCAGCAGTACGCACACA	

Supplementary References

1 Bonelli F, Sarasini A, Zierold C et al. Clinical and Analytical Performance of an Automated Serological Test That Identifies S1/S2-Neutralizing IgG in COVID-19 Patients Semiquantitatively, *J Clin Microbiol*. **58**(9): e01224–20 (2020)

2 Fraser C, Cummings DAT, Klinkenberg D et al. Influenza transmission in households during the 1918 pandemic, *Am J Epidemiol*. **174**(5):505–14 (2011).

3 Lloyd-Smith J, Schreiber SJ, Kopp PE and Getz WM, Superspreading and the effect of individual variation on disease emergence. *Nature* **438**: 355–359 (2005).

4 Lavezzo E, Franchin E, Ciavarella C *et al.* Suppression of a SARS-CoV-2 outbreak in the Italian municipality of Vo'. *Nature* **584,** 425–429 (2020).
