# Supplementary material for: SARS-CoV-2 antibody dynamics and transmission from community-wide serological testing in the Italian municipality of Vo’
Source: Nat Commun. 2021 Jul 19;12:4383. doi: 10.1038/s41467-021-24622-7 (PMC8289856; doi:10.1038/s41467-021-24622-7)
Supplement: Supplementary file 3 — Reporting Summary [file 41467_2021_24622_MOESM3_ESM.pdf]

nature research

Corresponding author(s): Dr Ilaria Dorigatti

Last updated by author(s): Jun 8, 2021

Reporting Summary

Nature Research wishes to improve the reproducibility of the work that we publish. This form provides structure for consistency and transparency in reporting. For further information on Nature Research policies, see our Editorial Policies and the Editorial Policy Checklist.

Statistics

For all statistical analyses, confirm that the following items are present in the figure legend, table legend, main text, or Methods section.

n/a	Confirmed	
☐	☒	The exact sample size (*n*) for each experimental group/condition, given as a discrete number and unit of measurement
☐	☒	A statement on whether measurements were taken from distinct samples or whether the same sample was measured repeatedly
☐	☒	The statistical test(s) used AND whether they are one- or two-sided<br>*Only common tests should be described solely by name; describe more complex techniques in the Methods section.*
☐	☒	A description of all covariates tested
☐	☒	A description of any assumptions or corrections, such as tests of normality and adjustment for multiple comparisons
☐	☒	A full description of the statistical parameters including central tendency (e.g. means) or other basic estimates (e.g. regression coefficient) AND variation (e.g. standard deviation) or associated estimates of uncertainty (e.g. confidence intervals)
☐	☒	For null hypothesis testing, the test statistic (e.g. *F*, *t*, *r*) with confidence intervals, effect sizes, degrees of freedom and *P* value noted<br>*Give P values as exact values whenever suitable.*
☐	☒	For Bayesian analysis, information on the choice of priors and Markov chain Monte Carlo settings
☐	☒	For hierarchical and complex designs, identification of the appropriate level for tests and full reporting of outcomes
☐	☒	Estimates of effect sizes (e.g. Cohen's *d*, Pearson's *r*), indicating how they were calculated

Our web collection on statistics for biologists contains articles on many of the points above.

Software and code

Policy information about availability of computer code

Data collection	Data were collated in Microsoft Excel.
Data analysis	We developed code in the R programming language (version 3.6.0) and in Python (version 3.7.3). We used the following R packages: binom, car, cowplot, data.table, dplyr, gdata, ggplot2, ggrepel, ggvenn, grid, gridExtra, gtable, odin, LaplacesDemon, stringr, tidyr, wesanderson, rmdformats, viridis. The code for the Metropolis-Hastings algorithm is implemented in function run_MCMC() in files: functions_fit_model.R, functions_fit_extended_model.R, functions_fit_2_groups_model.R and functions_model_11.R. The data and code are available at https://github.com/ncov-ic/Vo_serology.

For manuscripts utilizing custom algorithms or software that are central to the research but not yet described in published literature, software must be made available to editors and reviewers. We strongly encourage code deposition in a community repository (e.g. GitHub). See the Nature Research guidelines for submitting code & software for further information.

Data

Policy information about availability of data

All manuscripts must include a data availability statement. This statement should provide the following information, where applicable:

- Accession codes, unique identifiers, or web links for publicly available datasets
- A list of figures that have associated raw data
- A description of any restrictions on data availability

The dataset is available at https://github.com/ncov-ic/Vo_serology.

Field-specific reporting

Please select the one below that is the best fit for your research. If you are not sure, read the appropriate sections before making your selection.

- [x] Life sciences
- [ ] Behavioural & social sciences
- [ ] Ecological, evolutionary & environmental sciences

For a reference copy of the document with all sections, see nature.com/documents/nr-reporting-summary-flat.pdf

Life sciences study design

All studies must disclose on these points even when the disclosure is negative.

Sample size	The Vo' cluster (defined as residents of Vo' or nearby countryside settlements) comprises 3,329 subjects. We conducted a serological and nasopharyngeal swab survey in this population in May 2020, and tested 2,602 (78.2%) subjects. We found no swab positive subject and 162 (6.2%) subjects testing positive by at least one serological assay. In November 2020, we conducted a second serological and nasopharyngeal swab survey among the subjects who tested positive by May (either by PCR, any antibody assay or T-cells) and tested 156 subjects.<br>We did not conduct a sample size calculation and aimed to enroll as many subjects as possible.
Data exclusions	We did not exclude any data from the analysis.
Replication	We validated the sensitivity and specificity of the serological assays used in our analysis through in-house validation experiments, as reported in Padoan et al, EBioMedicine. 2020; 62:103101, https://doi.org/10.1016/j.ebiom.2020.103101. Detection of SARS-CoV-2 RNA was performed by an in-house real-time RT-PCR method performed at the Clinical Microbiology and Virology Unit of Padova University Hospital; we used the same method described in our previous study (Lavezzo et al., Nature, 2020, 584, 425–429, https://doi.org/10.1038/s41586-020-2956-7), which demonstrated 100% agreement with the assay validated by the National Reference Laboratory at the Italian Institute of Health (Istituto Superiore di Sanità). Each sample was analysed once with every serological and molecular assays.
Randomization	We conducted an observational study, randomization is not relevant. In the May 2020 survey, we aimed to enroll as many study participants as possible, regardless of the PCR results obtained in the previous surveys conducted in February and March 2020. In the November 2020 survey, we aimed to enroll as many PCR and/or seropositive subjects as possible.
Blinding	We conducted an observational study, blinding is not relevant.

Reporting for specific materials, systems and methods

We require information from authors about some types of materials, experimental systems and methods used in many studies. Here, indicate whether each material, system or method listed is relevant to your study. If you are not sure if a list item applies to your research, read the appropriate section before selecting a response.

Materials & experimental systems

n/a	Involved in the study	
x		Antibodies
	x	Eukaryotic cell lines
x		Palaeontology and archaeology
x		Animals and other organisms
	x	Human research participants
x		Clinical data
x		Dual use research of concern

Methods

n/a	Involved in the study	
x		ChIP-seq
x		Flow cytometry
x		MRI-based neuroimaging

Eukaryotic cell lines

Policy information about cell lines

Cell line source(s)	Vero cells were acquired directly from ATCC, RRID=CVCL_0574.
Authentication	No authentication procedures were performed outside the quality controls done by ATCC.
Mycoplasma contamination	The cell line was not tested for mycoplasma contamination (ATCC declares that contamination was not detected).
Commonly misidentified lines<br>(See ICLAC register)	n/a

Human research participants

Policy information about studies involving human research participants

Population characteristics	The covariate-relevant population characteristics of the human participants collected in the surveys were: age-class, sex, symptom occurrence, underlying health conditions, pharmacological therapy, hospitalization, household composition and contact network. The outcomes of interest were positivity to serological and PCR assays.
Recruitment	Participation was by consent; for subjects under the age of 18 years, consent was provided by a parent or legal guardian. For the first serosurvey, the blood and oro-nasopharyngeal samples were collected directly in Vo' over the weekend 1-3 May, 2020. The second serosurvey was performed on 28-29 November, 2020. Self-selection bias may have occurred due to the nature of the sampling procedure. Subjects participating to the follow-up serosurvey were summoned individually by telephone, recruiting only those who previously tested positive to at least a swab or a serological test.
Ethics oversight	The Ethics Committee for Clinical Research of the province of Padua approved the study.

Note that full information on the approval of the study protocol must also be provided in the manuscript.
